# Accelerated Intermittent Theta‐Burst Stimulation for the Treatment of Alcohol Use Disorder: A Randomized Sham‐Controlled Study With Six‐Month Follow‐Up

**DOI:** 10.1002/cns.71166

**Published:** 2026-09-16

**Authors:** Yunsheng Hou, Jingying Zhang, Shiyun Wang, Kunya Liu, Bingyu Zhang, Hongdu Deng, Rongrong Wang, Ying Xu, Dan Wang, Chuansheng Wang, Tianzhen Chen, Ruiling Zhang

**Affiliations:** ^1^ The Second Affiliated Hospital of Henan Medical University Xinxiang Henan China; ^2^ Henan Collaborative Innovation Center of Prevention and Treatment of Mental Disorder The Second Affiliated Hospital of Henan Medical University Xinxiang Henan China; ^3^ Department of Clinical Psychology, Central Institute of Mental Health University of Heidelberg/Medical Faculty Mannheim Mannheim Germany; ^4^ Shanghai Mental Health Center Shanghai Jiao Tong University School of Medicine Shanghai China

**Keywords:** accelerated iTBS, alcohol use disorder, craving, relapse

## Abstract

**Background:**

Accelerated intermittent theta‐burst stimulation (a‐iTBS) is a novel rTMS protocol increasingly used for mental disorders, but its application in alcohol use disorder (AUD) remains early. This exploratory sham‐controlled study evaluated a 50‐session a‐iTBS protocol for AUD.

**Methods:**

Sixty‐one male AUD inpatients were randomized to receive traditional iTBS (once daily, 600 pulse/session), a‐iTBS (5 sessions/day, 3000 pulses/day), or sham iTBS. Treatments were administered on 10 weekdays (Monday to Friday over 2 weeks) and targeted the dorsolateral prefrontal cortex. The primary outcome was the change in craving. The alcohol relapse behavior, emotional symptoms, and sleep quality were evaluated pre‐/post‐intervention.

**Results:**

Group showed no significant main effect on craving percentage change. The a‐iTBS group showed a higher response rate and earlier response onset than the s‐iTBS group, whereas no significant differences existed between a‐iTBS and iTBS, or iTBS and s‐iTBS. No intergroup differences were detected for mood and sleep indicators. At the 6‐month follow‐up, participants receiving a‐iTBS achieved longer abstinence duration than those given s‐iTBS. Exploratory logistic regression indicated that baseline GAD‐7 scores and treatment group were associated with treatment response.

**Conclusions:**

Although the primary craving outcome did not differ significantly among groups, a‐iTBS showed preliminary signals of earlier response and longer time to first relapse than sham stimulation. These exploratory findings require confirmation in larger trials.

## Graphical abstract

In this exploratory randomized sham‐controlled trial, accelerated iTBS was associated with a higher craving response rate and a faster initial response than sham stimulation in male inpatients with alcohol use disorder. These findings support further investigation of accelerated iTBS in larger and more diverse samples.

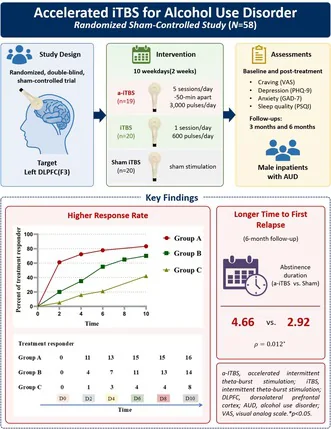

## 1 Background

Alcohol use disorder (AUD) is a chronically relapsing disorder with widespread global prevalence. According to the latest Global Status Report on Alcohol and Health and Treatment of Substance Use Disorders [1], approximately 17% of the world's population aged 15+ engaged in heavy episodic drinking or “binge drinking” and 7% met the criteria for AUD in 2019. In the meantime, 4.7% of all deaths were attributed to alcohol consumption. Despite the high prevalence and sever consequence, currently there is no universal effective treatment and only 0.3%–14% of people with AUD were in contact with treatment. Increasing endeavor has been put in developing and validating psychological interventions and pharmacotherapies. Among these, repetitive Transcranial Magnetic Stimulation (rTMS), a non‐invasive brain stimulation technique, has gained growing attention for its potential to modulate brain dysfunctions. RTMS is a U.S. FDA (Food and Drug Administration) approved treatment option for depression, while its efficacy in treating substance use disorders (SUD) has also be supported, with medium to large effect sizes reported in reducing craving and substance consumption [2, 3, 4, 5, 6]. The dorsolateral prefrontal cortex (dlPFC) is a well‐established target for treating depression and plays a fundamental role in executive control, emotion regulation, reward and motivation processes, as well as the transition from substance use to dependence [5, 7, 8], thus most studies have also targeted the dlPFC for rTMS in the treatment of SUDs [9]. Notably, a recent systematic review found that stimulation of the left dlPFC yields the most positive effects for various substances, except for alcohol, where mixed results have been reported [5].

Theta‐burst stimulation (TBS) is a patterned form of rTMS that mimics endogenous theta rhythms. Its after‐effects are thought to involve the modulation of synaptic plasticity, potentially through mechanisms such as NMDA receptor‐mediated glutamatergic transmission and BDNF expression [10, 11, 12, 13]. In line with common neurophysiological models, the intermittent Theta‐Burst Stimulation (iTBS) protocol used in this study is typically associated with increased cortical excitability (termed LTP‐like plasticity), while continuous TBS is associated with decreased excitability (LTD‐like plasticity) [14]. However, it is important to note that individual responses to these protocols can be highly variable, and the precise neurobiological mechanisms in humans remain an active area of research. Studies have demonstrated that a single session of iTBS can produce sustained enhancement of cortical excitability lasting up to 60 min, which has been associated with behavioral and therapeutic effects [15]. Increasing evidence supports the non‐inferiority of iTBS to established high‐frequency rTMS protocols in modulating depression related neural circuits [16, 17, 18, 19]. Owing to its shorter treatment duration, which often improves patient compliance, iTBS is considered a cost‐effective and efficient clinical treatment option. Its efficacy has also been explored in some other therapeutic indications, such as schizophrenia and SUD [20, 21]. A recent pilot study found that real iTBS targeting the left dlPFC significantly reduced relapse rates, anhedonia, and alcohol cue‐reactivity among veterans with AUD [22].

A more potent protocol, accelerated iTBS (a‐iTBS), has been developed to further improve the treatment efficacy, as increased neuromodulation dose is believed to enhance cortical excitability [23, 24]. A‐iTBS consists of multiple iTBS sessions per day with inter‐session intervals (e.g., 8–60 min), incorporating higher cumulative pulse dose. The increased number of pulses is hypothesized to induce cumulative and synergistic plastic changes, leading to more stable effects on cortical excitability. Accumulating evidence supports its safety, efficacy, and durability compared to conventional iTBS protocols, particularly in the treatment of depression [25, 26, 27, 28], while its application in AUD is still in early stages [29, 30].

In this randomized, sham‐controlled clinical trial, we evaluated the tolerability and efficacy of a 50‐session a‐iTBS protocol for inpatients with AUD. The a‐iTBS protocol consists of 3000 pulses stimulation per day, delivered in 5 sessions with a 50‐min inter‐session interval, over 10 weekdays (Monday to Friday) within a 2‐week period. We tracked the relapse status at 3 and 6 months after the intervention to assess the durability of its therapeutic effects. We hypothesized that, compared to the traditional forms of iTBS and the sham stimulation, a‐iTBS would be safe and tolerable, result in greater improvement in alcohol‐cue induced craving level, emotion and sleep quality after the treatment, prolong time to relapse, and reduce relapse rates during the follow‐up.

## 2 Methods

### 2.1 Participants and Procedure

Inpatients with AUD were recruited at the SUD ward of the Second Affiliated Hospital of Xinxiang Medical University from March 2022 to November 2023. Inclusion criteria: (1) met DSM‐5 criteria for AUD; (2) aged between 18 and 60 years; (3) fluency in Chinese; (4) right‐handed. Exclusion criteria: (1) Patients with severe physical diseases or cognitive impairment (such as head trauma, cerebrovascular disease, epilepsy, etc.) that cannot complete all interventions or tests; (2) Patients with (or have suffered from) other mental diseases (except nicotine use disorder); (3) Pregnant or lactating patients; (4) Patients taking medicines that affect cognitive function in the past 10 days; (5) Patients need to take antipsychotic medicines during the intervention; (6) Patients have received TMS or tDCS treatment before; (7) Patients have any contraindications to rTMS.

All participants provided written informed consent before enrolling in this study; they received compensation and were able to withdraw their consent at any time without negative consequences. All research procedures were conducted in accordance with the ethical principles outlined in the Declaration of Helsinki.

Sixty‐eight male participants were screened, of which 61 met the enrollment criteria and underwent randomization. All eligible subjects had passed the acute alcohol withdrawal phase and maintained alcohol abstinence for at least 7 days prior to enrollment. They were subsequently randomized into one of three groups to receive a‐iTBS (*N* = 21), iTBS (*N =* 20), or s‐iTBS (*N* = 20). Participants and outcome assessors were blinded to treatment allocation, whereas treatment operators were aware of the assigned stimulation condition but were not involved in outcome assessment or data analysis. Detailed randomization and blinding procedures are provided in the Supporting Information. All participants received a standardized inpatient treatment during the intervention period. This included pharmacological management with low‐dose benzodiazepines for withdrawal symptom control, nutritional support with vitamin supplementation, and structured psycho‐education.

**FIGURE 1:**
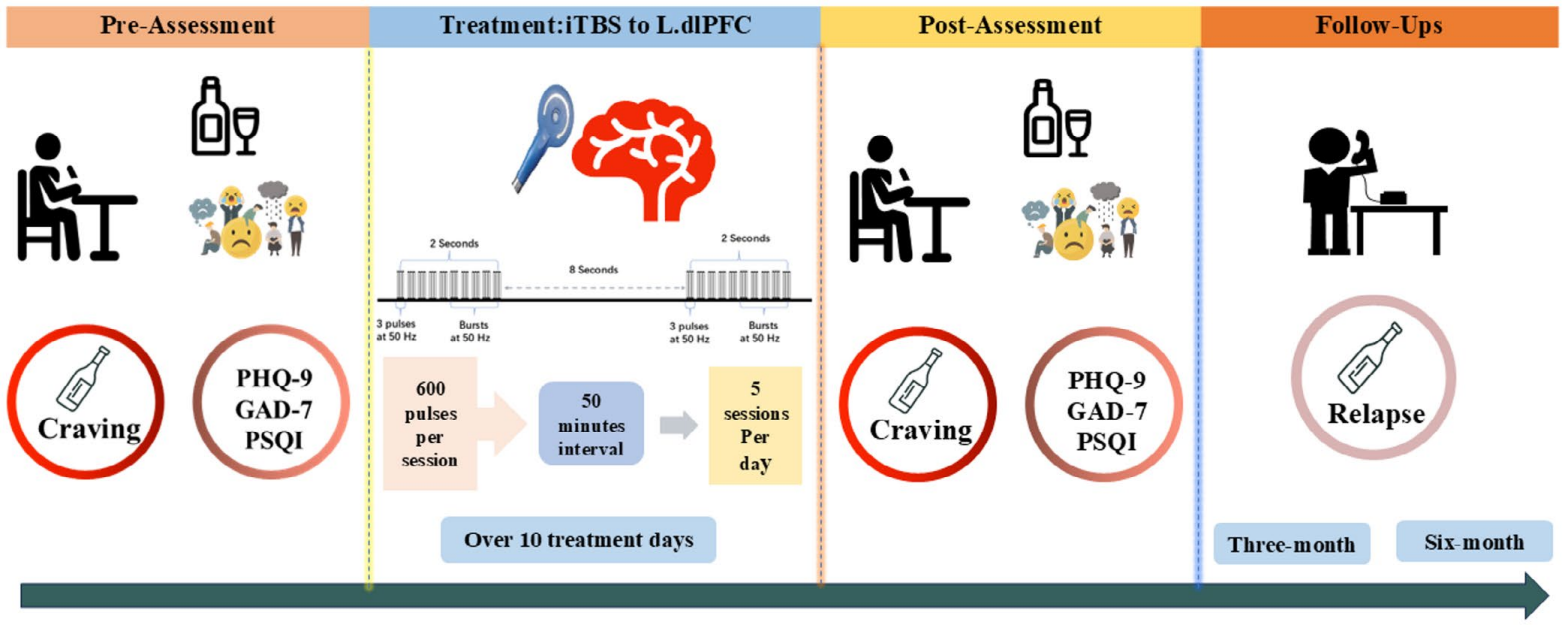
Schematic overview of the a‐iTBS procedure. The intervention procedure consists of four phases: Pre‐assessment, intervention, post‐intervention, and follow‐up. Participants were assessed for alcohol cue‐induced craving, emotional state, and sleep quality before and after the intervention. Craving was additionally evaluated every 2 days during the 10‐day intervention period. The a‐iTBS protocol consists of 3000 pulses stimulation per day, delivered in 5 sessions with a 50‐min inter‐session interval for 10 days. Participants were monitored for relapse at 3 and 6 months after the intervention.

**FIGURE 2:**
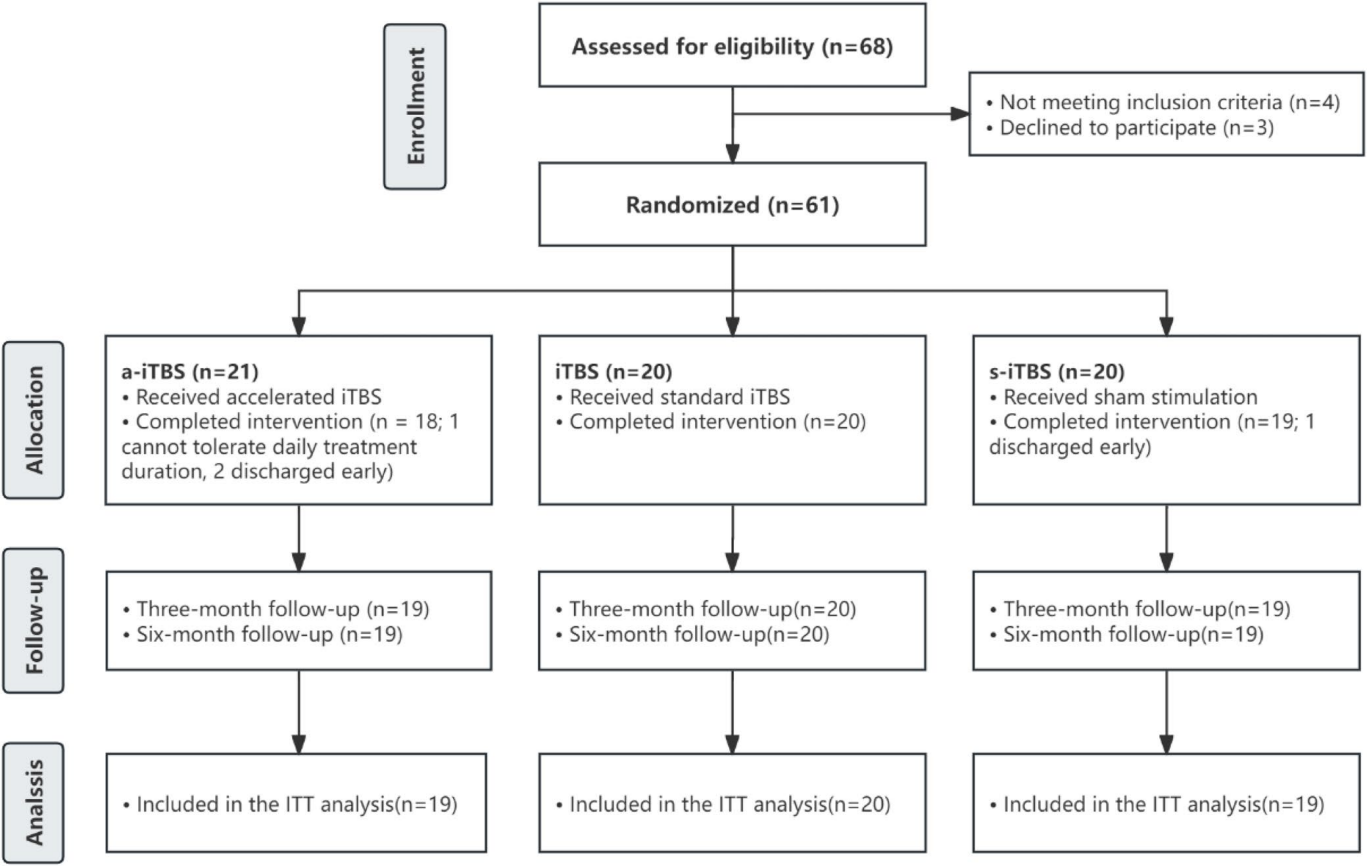
Flow chart. Flow diagram of the study procedure. Among the 61 randomized participants, 3 withdrew prior to baseline, and the final analytic sample consisted of 58 individuals.

After group allocation, participants took the baseline assessment. Following the baseline assessment, participants underwent their assigned intervention on 10 weekdays (Monday to Friday over 2 weeks). Side effects and tolerability were assessed and recorded on each intervention day. Craving was additionally assessed after treatment on intervention Days 2, 4, 6, 8, and 10. Three patients withdrew prematurely from the trial before undergoing any baseline assessments or interventions (2 in the a‐iTBS group and 1 in the s‐iTBS group). Additionally, one patient in the a‐iTBS group withdrew on the second day due to being unable to tolerate the daily treatment sessions. 61 AUD patients were assigned to receive one of these three interventions; 57 subjects completed the treatment. The primary analysis adhered to the modified intention‐to‐treat principle and included all randomized participants (*N* = 58) who completed the baseline assessment and received at least one intervention session. Participants were followed up at 3 and 6 months after the intervention; they were contacted via telephone to determine their relapse status (date of initial relapse) (Figures 1 and 2).

### 2.2 iTBS Intervention

The iTBS intervention was administered using the YRD CCY‐II magnetic stimulator (YiRuiDe Group, Wuhan, China). Stimulation targeted the left dlPFC, with the F3 electrode site as the target location identified according to the international 10–20 EEG system [31]. Prior to the intervention, the resting motor threshold (RMT) was determined for each participant by applying single pulse TMS to the left motor cortex with gradually increasing intensity. The RMT was defined as the minimum stimulus intensity required to induce right abductor pollicis brevis muscle contraction in at least 5 out of 10 trials [32]. The traditional iTBS protocol was employed, consisting of 3‐pulse 50‐Hz bursts repeated every 200 ms, with 2 s on and 8 s interval, lasting approximately 190 s with a total of 600 pulses [10]. Stimulation intensity was set at 70% of the RMT, in accordance with established safety protocols [33]. The standard iTBS group received one active iTBS session per day, corresponding to 600 pulses per day and 10 sessions in total. The a‐iTBS group received five active iTBS sessions per day, separated by approximately 50 min, corresponding to 3000 pulses per day and 50 sessions in total. Participants in the sham group were randomly assigned to receive sham stimulation delivered according to either the standard or accelerated schedule. For sham stimulation, the coil was rotated 90° perpendicular to the scalp to minimize the effective magnetic field [34].

### 2.3 Measurements

Baseline data on demographic characteristics (age and education) and alcohol use history (duration and frequency of alcohol use, average dose, detoxification period, and the number of inpatient detoxifications) were collected using a custom‐designed questionnaire and the Alcohol Use Disorders Identification Test (AUDIT) [35]. Our primary outcome is craving reduction percentage, assessed at baseline (D0) and immediately after TBS treatments on the 2nd, 4th, 6th, 8th, and 10th intervention days (D2, D4, D6, D8, D10) using the Visual Analogue Scale (VAS) [36]. To assess craving, participants were asked to view alcohol‐related images (presented via PowerPoint with a preset display time for 5 min), and then to place a mark on a 10‐cm line to indicate their craving, ranging from 0 (no craving) to 100 (extremely intense craving). The craving reduction rate was calculated as craving reduction percentage = [(craving_D0 − craving_D10)/craving_D0] × 100%, and the responder was defined as participants who achieved a reduction of 60% or more in craving scores [37]. The secondary outcome measures were the change from baseline to the end of intervention in anxiety, depression, and sleep quality, measured by the Generalized Anxiety Disorder‐7 (GAD‐7) scale [38], the Patient Health Questionnaire‐9 (PHQ‐9) [39], and the Pittsburgh Sleep Quality Index (PSQI) [40], respectively. Changes in secondary outcomes were calculated as Δ = post − pre. Finally, participants were followed up for a period of 6 months. At 3 and 6 months after the intervention, participants were contacted via phone and asked whether they had consumed alcohol, and if so, additional information was collected regarding the timing of relapse. This study primarily reports the cumulative abstinence duration (in months) from enrollment to the first relapse and the dichotomous relapse status at 3 and 6 months. Detailed randomization and blinding procedures, including the assessment of blinding integrity, are provided in the Supporting Information.

### 2.4 Data Analysis

Data analyses were performed using IBM SPSS Statistics 27 (IBM, Armonk, NY, USA). Baseline group differences were assessed using one‐way ANOVA for continuous variables and chi‐square (*χ* ^2^) tests for categorical variables. The primary analysis followed the modified intention‐to‐treat principle and included all randomized participants who completed the baseline assessment and received at least one intervention session (*N* = 58). The effect of group on craving reduction percentage was examined using a linear mixed model, with group as a fixed effect, participant ID as a random intercept, and age, cumulative drinking years, AUDIT total score, and number of prior inpatient detoxifications as covariates. Multicollinearity was assessed. Missing data were handled using multiple imputation, and pooled estimates were reported. Changes in PHQ‐9, GAD‐7, and PSQI scores were analyzed using analogous models adjusted for their respective baseline values. Time to initial response and time to first relapse were analyzed using Kaplan–Meier survival analyses and log‐rank tests. For the relapse analyses, participants without relapse were censored at the end of the corresponding follow‐up period, whereas those with incomplete follow‐up were censored at their last known contact. Pearson correlations examined associations between craving reduction and secondary clinical outcomes. Multiple linear regression was used to identify factors associated with craving reduction, and logistic regression was used to examine factors associated with treatment response. These predictor analyses were considered exploratory. Statistical significance was set at *p* < 0.05. Bonferroni correction was applied to prespecified pairwise group comparisons within each outcome and follow‐up time point. Effect sizes were reported as partial eta squared (*ηp* ^2^) for omnibus *F* tests and *φ* coefficients for pairwise chi‐square and log‐rank comparisons. Odds ratios with 95% confidence intervals were reported for logistic regression analyses.

## 3 Results

There were no significant differences between the randomized groups in demographics and alcohol use characteristics, but craving scores (*p* = 0.006), GAD‐7 (*p* = 0.031), and PSQI scores (*p* = 0.030) varied among the three groups (Table 1). Baseline demographics, alcohol use characteristics, and clinical variables are presented in Table 1. The intervention was safe and well tolerated, with no serious adverse events reported. Side effects experienced by participants included headache, drowsiness, facial discomfort, eye and nasal discomfort, and jaw or tooth discomfort. All events were mild, short‐lived, and resolved spontaneously. There was no significant difference in the incidence of adverse reactions among the three groups (*p* = 0.515). Participants' guesses were not significantly associated with their actual treatment allocation (*χ* ^2^ = 0.138, *p* = 0.933). Outcome assessors correctly classified the treatment condition for 30 of 58 participants (51.7%), which did not differ significantly from the 50% chance level (exact binomial test, *p* = 0.896). Detailed results are provided in the Supporting Information.

**TABLE 1:** Baseline demographics, alcohol use characteristics and clinical variables.

	a‐iTBS	iTBS	s‐iTBS	*F*/*χ* ^2^	*p*
*n*	19	20	19		
Age, mean ± SD	36.05 ± 7.15	38.5 ± 8.20	37.26 ± 7.45	0.502	0.608
Years of education, mean ± SD	11.05 ± 3.33	10.90 ± 3.26	10.79 ± 3.15	0.033	0.968
Accumulated years of alcohol use, mean ± SD	16.53 ± 6.98	18.80 ± 8.94	17.32 ± 8.45	0.390	0.679
Average frequency, day, mean ± SD	22.32 ± 9.19	25.20 ± 9.40	25.79 ± 5.07	0.990	0.378
Average dose, mL, mean ± SD	347.37 ± 198.94	577.50 ± 515.66	468.42 ± 216.16	2.149	0.126
Detoxification, month, mean ± SD	0.79 ± 0.54	1.10 ± 0.44	0.89 ± 0.31	2.499	0.091
Number of inpatient detoxifications, mean ± SD	1.68 ± 2.21	1.15 ± 2.25	1.63 ± 3.18	0.255	0.776
Benzodiazepines dose, mg/day, mean ± SD	14.60 ± 6.83	13.87 ± 8.52	13.02 ± 6.04	0.23	0.79
AUDIT, mean ± SD	24.26 ± 5.89	28.15 ± 6.12	25.95 ± 6.08	2.040	0.140
Craving, mean ± SD	22.11 ± 22.50	29.50 ± 27.23	52.11 ± 35.52	5.573	0.006
PHQ‐9, mean ± SD	11.53 ± 7.74	7.45 ± 5.24	6.37 ± 7.55	2.954	0.060
GAD‐7, mean ± SD	7.68 ± 6.83	2.65 ± 3.12	5.05 ± 6.73	3.692	0.031
PSQI, mean ± SD	9.21 ± 5.77	5.90 ± 3.37	5.57 ± 4.23	3.736	0.030

Abbreviations: a‐iTBS, accelerated intermittent theta‐burst stimulation; AUDIT, Alcohol Use Disorders Identification Test; GAD‐7, general anxiety disorder‐7; iTBS, intermittent theta‐burst stimulation; PHQ‐9, Patient Health Questionnaire‐9; PSQI, Pittsburgh Sleep Quality Index; s‐iTBS, sham iTBS; SD, standard deviation.

Considering that participants in the sham group actually received one of two different schedules (matched to the a‐iTBS or standard iTBS protocol, respectively), we conducted a sensitivity analysis to evaluate whether the difference in schedules introduced bias. The two sham subgroups did not differ significantly on any primary or secondary outcome, supporting their pooling into a single sham group for the main analyses. Detailed results are presented in Table S1. To address baseline imbalances in craving, GAD‐7, and PSQI scores, we repeated the primary analysis with these three baseline scores entered as additional covariates; the adjusted results remained consistent with the main findings: adjusted results are presented in Table S2.

### 3.1 Primary Outcome Measure

After controlling for covariates using a linear mixed model, the main effect of group on the percentage change in craving was not statistically significant (*F* (2, 71.65) = 2.69, *p* = 0.074, *ηp* ^2^ = 0.07). The number of hospitalizations for alcohol abstinence had a significant negative effect on the percentage change in craving (*β* = −12.10, *t* (71.65) = −2.56, *p* = 0.010, *r* = 0.29). Furthermore, no other covariates in the model showed significant effects. Consistent with this, linear regression analysis (*F* = 2.470, *p* = 0.036) indicated that a lower number of prior inpatient alcohol treatments (*t* = −1.96, *p* = 0.049) was associated with a greater percentage reduction in craving, while other variables were not significant predictors.

**FIGURE 3:**
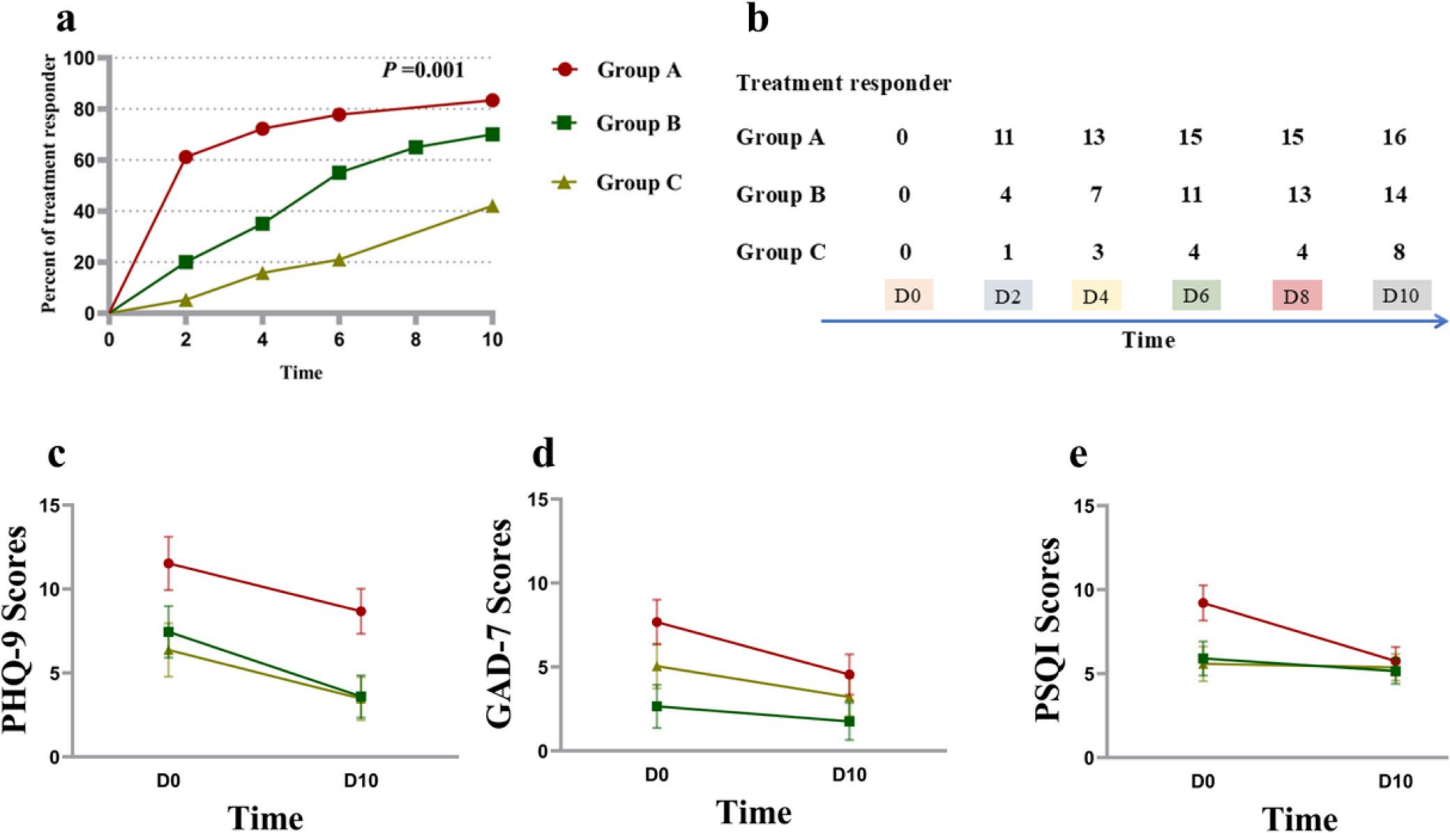
Changes in the primary outcomes and secondary outcomes. (a) The rate and (b) the number of responders in the three groups during the intervention period. (c) PHQ‐9, (d) GAD‐7, and (e) PSQI from baseline (D0) to the end of the intervention (D10). Error bars represent the standard errors. Group A: A‐iTBS, Group B: ITBS, Group C: S‐iTBS.

The response rate was defined as the proportion of participants achieving a ≥ 60% reduction in craving scores. Response rates were 84.2%, 70.0%, and 42.1% in the a‐iTBS, iTBS, and s‐iTBS groups, respectively. The overall difference among the three groups was significant (*χ* ^2^ = 7.726, df = 2, *p* = 0.021). In pairwise comparisons, the response rate was significantly higher in the a‐iTBS group than in the s‐iTBS group (*χ* ^2^ = 7.238, df = 1, *p* = 0.007, *φ* = 0.377), whereas no significant differences were observed between the a‐iTBS and iTBS groups (*χ* ^2^ = 1.108, df = 1, *p* = 0.292, *φ* = 0.169) or between the iTBS and s‐iTBS groups (*χ* ^2^ = 3.083, df = 1, *p* = 0.079, *φ* = 0.281). Kaplan–Meier analysis of time to initial response showed a significant overall difference among the three groups (log‐rank *χ* ^2^ = 13.247, df = 2, *p* = 0.001). Bonferroni‐adjusted post hoc pairwise comparisons showed that the time to response was significantly shorter in the a‐iTBS group than in the s‐iTBS group (log‐rank *χ* ^2^ = 12.059, df = 1, *p* < 0.001, *φ* = 0.563), whereas the differences between the iTBS and s‐iTBS groups (log‐rank *χ* ^2^ = 4.405, df = 1, *p* = 0.108, *φ* = 0.336) and between the a‐iTBS and iTBS groups (log‐rank *χ* ^2^ = 3.214, df = 1, *p* = 0.219, *φ* = 0.287) were not significant (Table 2, Figure 3).

**TABLE 2:** Changes in primary and secondary outcomes.

	a‐iTBS	iTBS	s‐iTBS	*F*/*χ* ^2^	*p*	Group difference
*n*	19	20	19			
Primary outcomes						
% Craving reduction, mean ± SD	78.27 ± 38.59	27.10 ± 131.70	22.08 ± 111.17	2.690	0.074	
Exploratory craving‐response outcomes						
Responder, %	84.2%	70.0%	42.1%	7.726	0.021	a‐iTBS > s‐iTBS
Response time, days, mean ± SE	4.12 ± 0.76	6.50 ± 0.72	8.74 ± 0.62	13.247	0.0013	a‐iTBS > s‐iTBS
Secondary outcomes						
ΔPHQ‐9, mean ± SD	5.70 ± 6.55	3.85 ± 4.56	2.89 ± 4.12	1.690	0.199	
ΔGAD‐7, mean ± SD	3.31 ± 5.68	0.90 ± 4.28	1.84 ± 5.09	0.045	0.956	
ΔPSQI, mean ± SD	3.47 ± 3.81	0.75 ± 2.94	0.21 ± 3.03	0.568	0.574	
Three‐month follow‐up: *n* = 19/20/19						
Relapse drinking, %	36.8%	60.0%	63.2%	3.171	0.205	
Abstinence duration, months, mean ± SE	2.72 ± 0.17	2.35 ± 017	2.00 ± 0.20	4.720	0.094	
Six‐month follow‐up: *n* = 19/20/19						
Relapse drinking, %	42.1%	60.0%	73.7%	3.930	0.140	
Abstinence duration, months, mean ± SE	4.66 ± 0.44	3.68 ± 0.45	2.92 ± 0.44	6.56	0.038	a‐iTBS > s‐iTBS

*Note:* Craving reduction percentage = [(craving_D0 − craving_D10)/craving_D0 × 100%], responder: the percentage of participants achieving ≥ 60% reduction on craving scores, ΔPHQ‐9 = PHQ‐9_post‐PHQ‐9_pre, ΔGAD‐7 = GAD‐7_post‐GAD‐7_pre, ΔPSQI = PSQI_post‐PSQI_pre.

Additionally, an exploratory logistic regression analysis (AUC = 0.89, 95% CI = 0.81–0.98; sensitivity = 0.84; specificity = 0.84) identified higher baseline GAD‐7 scores (OR = 1.55, 95% CI = 1.09–2.22, *p* = 0.015) and treatment group as significant predictors of treatment response after 10 days of iTBS treatment. Specifically, participants in the a‐iTBS group were more likely to respond compared to those in the s‐iTBS, as were those in the iTBS group compared to the s‐iTBS group (a‐iTBS vs. s‐iTBS: OR = 15.01, 95% CI: 1.56–144.78, *p* = 0.019; iTBS vs. s‐iTBS: OR = 12.11, 95% CI: 1.36–107.67, *p* = 0.025), whereas no significant difference was found between the a‐iTBS and iTBS groups (OR = 1.10, 95% CI: 0.69–1.77, *p* = 0.685) (Table 3).

**TABLE 3:** Logistic regression analysis of variables predicting intervention response.

	*B*	SE	Wald	df	*p*	OR	95% CI for OR	
							Lower	Upper
Baseline GAD scores	0.44	0.18	5.91	1	0.015	1.55	1.09	2.22
Groups (Reference: s‐iTBS)			6.94	2	0.031			
a‐iTBS and s‐iTBS	2.71	1.16	5.49	1	0.019	15.01	1.56	144.78
iTBS and s‐iTBS	2.49	1.12	5.00	1	0.025	12.11	1.36	107.67

### 3.2 Secondary Outcome Measures

Kaplan–Meier survival analysis was used to evaluate the time to first relapse (in months), with group comparisons performed using the log‐rank test and pairwise comparisons adjusted using the Bonferroni correction. All analyses adhered to the modified intention‐to‐treat principle; participants who completed follow‐up without relapse, those lost to follow‐up, and those who withdrew early were censored at their last known contact time. At the 3‐month follow‐up, the overall survival curves did not differ significantly among the three groups (log‐rank *χ* ^2^ = 4.72, *p* = 0.094). The mean survival times (months) were 2.72 ± 0.17 (95% CI 2.39–3.05) for the a‐iTBS group, 2.35 ± 0.17 (95% CI 2.02–2.68) for the iTBS group, and 2.00 ± 0.20 (95% CI 1.61–2.39) for the sham group. At the 6‐month follow‐up, the overall survival curves differed significantly among the three groups (log‐rank *χ* ^2^ = 6.56, *p* = 0.038). The mean survival times were 4.66 ± 0.44 (95% CI 3.80–5.51) for the a‐iTBS group, 3.68 ± 0.45 (95% CI 2.80–4.56) for the iTBS group, and 2.92 ± 0.44 (95% CI 2.06–3.78) for the sham group. Bonferroni‐adjusted pairwise comparisons showed that the a‐iTBS group had a significantly longer mean survival time than the sham group (*p* = 0.012), whereas no significant differences were found between the a‐iTBS and iTBS groups (*p* = 0.122) or between the iTBS and sham groups (*p* = 0.265). While the relapse rates did not differ among the three groups (Table 2, Figure 3). The analysis of other secondary clinical outcomes did not differ between the three groups. Pearson correlation analysis revealed a significant positive correlation between the craving reduction percentage and the change in GAD‐7 scores (*r* = 0.310, *p* = 0.018), while no significant correlations were observed for other secondary variables.

## 4 Discussion

This exploratory randomized controlled trial aimed to investigate the intervention effects of a‐iTBS on craving, emotional status, and long‐term relapse among inpatients with alcohol use disorder (AUD). No significant between‐group differences were found regarding the degree of craving alleviation. Only subtle favorable trends of faster craving alleviation and prolonged abstinence were observed in the a‐iTBS group relative to the sham stimulation group, while no intergroup benefits were found for depression, anxiety, or sleep outcomes. Collectively, a‐iTBS failed to demonstrate overall superior therapeutic effects compared with standard iTBS and sham stimulation.

Craving represents a critical outcome measure in alcohol addiction research. The present study detected no significant effects of 10 sessions of conventional iTBS or 50‐session accelerated iTBS (a‐iTBS) targeting the left dorsolateral prefrontal cortex (DLPFC) on acute alcohol craving, despite subtle favorable trends in response rate and onset speed observed in the a‐iTBS group. These findings align with several prior investigations that failed to identify robust craving‐lowering effects of high‐frequency repetitive transcranial magnetic stimulation (HF‐rTMS) among individuals with alcohol use disorder (AUD). One small‐scale RCT (*n* = 19) reported comparable alcohol craving between 10 sessions of 20 Hz HF‐rTMS over the left DLPFC and sham stimulation, with no intergroup disparities [41]. Another single‐blind, sham‐controlled RCT enrolling 60 male inpatients also demonstrated that 10 sessions of 10 Hz HF‐rTMS targeting the left DLPFC exerted no meaningful impacts on craving [42]. A larger inpatient RCT (*n* = 80) utilizing 10 Hz stimulation (3000 pulses per day for 10 days) over the right DLPFC similarly yielded null effects on acute craving [43]. Two recent randomized, double‐blind, sham‐controlled trials adopting iTBS protocols replicated these neutral results: both real and sham stimulation groups exhibited significant within‐group craving reductions over time, yet no incremental craving‐lowering benefits were attributed to active iTBS [22, 44].

Contrary to the aforementioned null findings, several studies have yielded positive results regarding the craving‐suppressing effects of rTMS. A sham‐controlled trial (*n* = 45) demonstrated that 10 sessions of 10 Hz high‐frequency rTMS delivered over the right DLPFC significantly reduced alcohol craving [45]. A subsequent randomized double‐blind trial (*n* = 20) comparing the anti‐craving efficacy of left versus right rTMS found robust within‐group craving reductions following stimulation on both sides, with no meaningful difference in therapeutic effects between left and right targets [46]. Another RCT targeting the left DLPFC (*n* = 45) also reported marked alleviation of alcohol craving after 10 sessions of 20 Hz high‐frequency rTMS [47]. Additionally, a small‐scale RCT (*n* = 30) observed sustained craving attenuation for up to 3 months after 10 sessions of 10 Hz high‐frequency rTMS (3000 pulses per day) applied to the right DLPFC [48].

Substantial heterogeneity exists in previous evidence regarding relapse outcomes following rTMS for alcohol dependence. One study administering 10 sessions of high‐frequency rTMS over the right DLPFC reported a 1‐month relapse rate of 13.8% in the active group versus 33.3% in the sham group, yet this numerical gap failed to reach statistical significance [45]. Another trial with identical right DLPFC targeting and 10 stimulation sessions (30,000 total pulses) noted superior reductions in drinking quantity and heavy drinking days within the active arm over 12 months of follow‐up, though abstinence rates at endpoint were comparable across groups, and therapeutic effects waned after the third month [48]. By contrast, a large‐scale RCT (*n* = 80) adopting comparable stimulation parameters detected no significant between‐group differences in 6‐month abstinent days, time to relapse, or continuous abstinence rates [43]. A deep rTMS trial targeting the insula likewise yielded no meaningful improvements in drinking behaviors [49]. For left DLPFC stimulation, one trial demonstrated a significantly higher 6‐month continuous abstinence rate after 20 sessions of iTBS (24,000 pulses) relative to sham (59% vs. 41%) [44]. A preliminary study similarly reported 3‐month relapse rates of 20% in the active group and 75% in the sham group. A multicenter RCT further found that left DLPFC rTMS combined with standardized cognitive behavioral therapy (CBT) produced markedly lower relapse rates compared with sham plus CBT (22.2% vs. 45.0%) [50]. Nevertheless, evidence for relapse mitigation via left DLPFC stimulation remains inconsistent: one investigation found no intergroup disparities in alcohol consumption at the 1‐month follow‐up [51], while two additional studies reported null effects on craving, albeit without direct relapse analyses [41, 42].

The exact drivers underlying such heterogeneous findings remain unclear. Notably, nearly all trials documenting favorable relapse‐related outcomes delivered a minimum of 10 stimulation sessions with total pulses ≥ 30,000 or equivalent dosages, and most targeted the left DLPFC [22, 44, 48], implying that sufficient stimulation dosage and target location may contribute to the heterogeneity across studies, although their respective effects cannot be determined from the currently available evidence. As a preliminary exploratory trial with a limited sample exclusively comprising male inpatients, the observed trend toward reduced relapse in the present study warrants validation in larger RCTs with extended follow‐up durations. Uniform consensus on standardized definitions of successful AUD treatment has not yet been established [52]. Future investigations should integrate metrics including relapse severity and alcohol intake reduction into relapse assessment batteries, and systematically dissect the respective impacts of distinct stimulation targets, paradigms, and total pulse dosages on relapse‐related endpoints [41, 49, 50, 53, 54]. One trial utilizing the HDRS and BDI identified no meaningful intergroup discrepancies between active and sham stimulation arms [41]. Another study enrolling 39 individuals with AUD similarly failed to detect additional benefits of rTMS relative to sham stimulation [53]. A deep rTMS trial targeting the insula only revealed significant temporal changes rather than between‐group differences in depression and anxiety subscale scores [49]. Analogous neutral outcomes were also documented in deep TMS protocols directed at the medial prefrontal cortex and a large multicenter RCT [50, 54].

These collective observations suggest that improvements in emotional and sleep outcomes may have been related to comprehensive inpatient care, spontaneous improvement over time, or other non‐specific therapeutic factors, rather than being attributable specifically to rTMS. Although changes in anxiety, depression, and sleep outcomes did not differ significantly among the treatment groups, higher baseline GAD‐7 scores were associated with a greater likelihood of meeting the craving‐response criterion in an exploratory multivariable logistic regression analysis. This association was observed across the overall sample and should not be interpreted as evidence that iTBS, or a‐iTBS specifically, was more effective in participants with higher anxiety. Because no prespecified anxiety subgroup or treatment‐by‐anxiety interaction analysis was conducted, and given the limited sample size, this exploratory finding requires confirmation in larger studies.

This study has several limitations. First, although the study was preregistered, some deviations from the original registration occurred. The planned target sample size was not fully achieved because recruitment proceeded more slowly than anticipated and was partly affected by COVID‐19‐related disruptions during the early recruitment period. The final analytic sample of 58 participants, with 19–20 participants per group, limited the statistical power to detect smaller but potentially clinically meaningful between‐group differences. In addition, all participants were male inpatients. Therefore, potential gender differences could not be examined, and the findings may not generalize to female patients, outpatients, or broader populations with AUD. Future multicenter studies with larger and more diverse samples, including female participants, are needed to confirm the present findings. In addition, although neuroimaging was part of the preregistered protocol, this component could not be completed due to technical constraints. And because of that, present study cannot establish target engagement or determine the neural mechanisms underlying the observed clinical findings. Second, participants continued benzodiazepines administration during the intervention period to avoid disrupting their standard inpatient care, while prior study suggests that benzodiazepines may affect the efficacy of TMS [42]. Nevertheless, the administration dosage did not differ among the three groups, which may have minimized the potential confounding effects. However, medication dosage and duration were not recorded repeatedly during the intervention, its potential influence on the outcomes could therefore not be fully evaluated. Third, we delivered the sham stimulation by tilting the coil 90 degrees perpendicular to the scalp, which could potentially be recognized by participants. However, post‐intervention interviews indicated that participants had difficulty distinguishing between real and sham stimulation. Future studies may consider using dedicated sham coils to reduce this potential source of bias. Finally, although participants were randomly assigned to intervention groups, baseline craving, GAD‐7, and PSQI scores differed between the groups. We believe this imbalance occurred by chance due to the randomization procedure, which is more likely in studies with relatively small sample sizes. Additional covariate‐adjusted analyses were therefore conducted, but residual confounding cannot be completely excluded.

## 5 Conclusions

In this exploratory pilot RCT, a‐iTBS appeared safe and tolerable in inpatients with AUD, showing preliminary signals of faster craving reduction and higher response rates versus sham. However, pairwise comparisons for the primary craving outcome were non‐significant, and the small sample limits definitive conclusions. These findings are hypothesis‐generating and require confirmation in larger, adequately powered trials.

## Author Contributions

Y.H. and J.Z. contributed equally to this work and share first authorship. J.Z., T.C., and R.Z. conceived the study protocol. Y.H. performed the intervention, analyzed the data, interpreted the results, and contributed to manuscript writing. J.Z. designed the study protocol, registered the study, prepared experimental materials, and drafted the manuscript. S.W., K.L., B.Z., H.D., R.W., and Y.X. assisted with data collection. D.W. and C.W. provided clinical support in the ward. T.C. and R.Z. supervised the project, revised the manuscript, and provided senior guidance. All authors reviewed and approved the final manuscript for submission.

## Funding

This research was supported by the Open Project of Psychiatry and Neuroscience Discipline of Second Affiliated Hospital of Xinxiang Medical University (2021‐zxkfkt‐001), National Natural Science Foundation of China (82571704, 82201650), Shanghai “Rising Stars of Medical Talents” Youth Development Program‐Specialist Program (SHWSRS(2025)_071), Shanghai Rising‐star Cultivation Program (22YF1439200), Clinical Research Project of Shanghai Municipal Health Commission (20244Y0201), Capability Promotion Project for Research‐oriented Doctor at SMHC (2021‐YJXYS‐01), Research Project of National Center for Mental Health of China (XS24B072), Key Research Project Plan for Higher Education Institutions in Henan Province (Project No.: 24A320014).

## Ethics Statement

The study protocol was reviewed and approved by the Ethics Committee of the Second Affiliated Hospital of Xinxiang Medical University (XYEFYLL‐2022‐03) and registered at the Chinese Clinical Trial Registry (ChiCTR2100043824).

## Consent

All participants provided written informed consent and were able to withdraw their consent at any time without negative consequences.

## Conflicts of Interest

The authors declare no conflicts of interest.

## Supporting information


**Appendix S1:** Supplementary methods and analyses, including randomization and blinding procedures, sensitivity power analysis, and additional sensitivity analyses.
**Table S1:** Comparison of baseline characteristics and clinical outcomes between the two sham stimulation schedules.
**Table S2:** Comparison of primary covariate‐adjusted and additionally adjusted linear mixed models for primary and secondary outcomes.

## Data Availability

The dataset generated and analyzed during the current research are not publicly available due to data protection laws but are available from the corresponding author on reasonable request.
