# Supplementary material for: Accelerated Intermittent Theta‐Burst Stimulation for the Treatment of Alcohol Use Disorder: A Randomized Sham‐Controlled Study With Six‐Month Follow‐Up
Source: CNS Neurosci Ther. 2026 Sep 16;32(9):e71166. doi: 10.1002/cns.71166 (PMC13580346; doi:10.1002/cns.71166)
Supplement: Supplementary file 1 [file CNS-32-e71166-s001.docx]

**Appendix S1. Supplementary Information**

Additional information on randomization and blinding, sensitivity power analyses, sham subgroup analyses, and adjusted analyses of primary and secondary outcomes.

**Contents of the Supplementary Material:**

Randomization and Blinding Procedures

Sensitivity power analysis for the planned sample size

Sensitivity Analysis of Sham Subgroups

Comparison of unadjusted and adjusted linear mixed models for primary and secondary outcomes.

**Randomization and Blinding**

Participants were randomly assigned in a 1:1:1 ratio to the a-iTBS, standard iTBS, or sham stimulation group using a computer-generated randomization sequence. Allocation concealment was ensured using sequentially numbered, opaque, sealed envelopes prepared and maintained by an independent researcher. The envelopes were opened by the study coordinator only after participant enrollment had been completed. The same stimulation device was used across conditions. However, potential differences in scalp sensations between active and sham stimulation could not be completely excluded.

Blinding integrity was evaluated after completion of the intervention. Participants and outcome assessors independently completed questionnaires and were asked to judge whether active or sham stimulation had been administered. Among participants, 89.5% (17/19) in the a-iTBS group, 90.0% (18/20) in the standard iTBS group, and 94.7% (18/19) in the sham group believed that they had received active stimulation. The distribution of guesses did not differ significantly among the treatment groups (χ² = 0.138, p = 0.933). Outcome assessors correctly classified the treatment condition for 30 of 58 participants (51.7%), which did not differ significantly from the 50% chance level (exact binomial test, p = 0.896). These results suggest that participant and outcome-assessor blinding was generally maintained.

**Sensitivity power analysis for the planned sample size**

The published study protocol prespecified a target sample size of 150 participants, with 50 participants in each of the three groups, but did not report the statistical assumptions underlying this target. To quantitatively evaluate the planned sample size, a sensitivity power analysis was conducted using G*Power 3.1 [1]. For a three-group omnibus one-way analysis of variance, with α = 0.05, 80% power, and a total sample size of 150, the minimum detectable effect size was Cohen’s f = 0.26. Thus, the prespecified target sample size would have provided adequate power to detect an approximately medium overall group effect.

**Sensitivity Analysis of Sham Subgroups**

Supplementary Table 1. Comparison of baseline characteristics and clinical outcomes between the two sham stimulation schedules.

|  | Sham‑a | Sham‑i | Statistical Comparison |
| --- | --- | --- | --- |
| Sample siz | 9 | 10 |  |
| Age, mean ± SD | 35.00±7.66 | 39.30±7.00 | *F* =1.632, *P* = 0.219 |
| Years of education, mean ± SD | 10.78±2.77 | 10.80±3.22 | *F* = 0.000, *P* = 0.987 |
| Accumulated years of alcohol use, mean ± SD | 16.11±9.76 | 18.40±7.42 | *F* = 0.335, *P* = 0.570 |
| Average frequency, day, mean ± SD | 25.56±5.27 | 26.00±5.16 | *F* = 0.034, *P* = 0.855 |
| Average dose, ml, mean ± SD | 444.44±98.24 | 490.00±289.44 | *F* = 0.201, *P* = 0.660 |
| Detoxification, month, mean ± SD | 1.00±0.00 | 0.80±0.42 | *F* = 2.013, *P* = 0.174 |
| Number of inpatient detoxifications, mean ± SD | 0.89±1.69 | 2.30±4.08 | *F* = 0.927, *P* = 0.349 |
| AUDIT, mean ± SD | 24.77±6.18 | 27.00±6.12 | *F* = 0.618, *P* = 0.443 |
| Craving-baseline, mean ± SD | 62.22±29.05 | 43.00±39.73 | *F* = 1.419, *P* = 0.250 |
| PHQ-9-baseline, mean ± SD | 4.33±6.59 | 8.20±8.21 | *F* = 1.260, *P* = 0.277 |
| GAD-7-baseline, mean ± SD | 2.77±5.80 | 7.10±7.12 | *F* = 2.071, *P* = 0.168 |
| PSQI-baseline, mean ± SD | 5.66±4.27 | 5.50±4.42 | *F* = 0.007, *P* = 0.935 |
| % craving reduction, mean ± SD | 27.85±44.47 | 16.88±151.32 | *F* = 0.013, *P* = 0.912 |
| Responder, % | 22.2% | 60% | χ2 = 2.773, *P* =0.096 |
| Respond time, days, mean ± SE | 8.89±0.71 | 8.60±0.98 | χ2 = 1.933, *P* =0.164 |
| ΔPHQ-9, mean ± SD | 1.33±2.64 | 4.30±4.80 | *F* = 1.045, *P* = 0.327 |
| ΔGAD-7, mean ± SD | 0.88±1.61 | 2.70±6.91 | *F* = 0.159, *P* = 0.697 |
| ΔPSQI, mean ± SD | 0.88±3.75 | -0.40±2.22 | *F* = 0.176, *P* = 0.682 |
| Three-month follow-up | | | |
| Relapse drinking, % | 66.7% | 60% | χ2 = 0.090, *P* =0.764 |
| Abstinence duration, months, mean ± SE | 2.00±0.272 | 2.00±0.283 | χ2 = 0.017, *P* =0.895 |
| Six-month follow-up | | | |
| Relapse drinking, % | 66.7% | 80% | χ2 = 0.434, *P* =0.510 |
| Abstinence duration, months, mean ± SE | 3.14±0.70 | 2.75±0.53 | χ2 = 0.237, *P* =0.626 |

Sham-a, sham stimulation matched to the accelerated iTBS schedule; Sham-i, sham stimulation matched to the standard iTBS schedule. AUDIT, Alcohol Use Disorders Identification Test; PHQ-9, Patient Health Questionnaire-9; GAD-7, Generalized Anxiety Disorder-7; PSQI, Pittsburgh Sleep Quality Index.

**Comparison of unadjusted and adjusted linear mixed models for primary and secondary outcomes.**

Supplementary Table 2. Comparison of primary covariate-adjusted and additionally adjusted linear mixed models for primary and secondary outcomes.

| Outcome | Model | Main Effect of Group |
| --- | --- | --- |
| Craving reduction (%) | Unadjusted | F= 2.690, *p* = 0.074 |
| Craving reduction (%) | Adjusted | F = 0.667, *p* = 0.520 |
| ΔPHQ-9 | Unadjusted | F = 1.690, *p* = 0.199 |
| ΔPHQ-9 | Adjusted | F = 0.697, *p* = 0.498 |
| ΔGAD-7 | Unadjusted | F = 0.045, *p* = 0.956 |
| ΔGAD-7 | Adjusted | F = 0.086, *p* = 0.918 |
| ΔPSQI | Unadjusted | F = 0.568, *p* = 0.574 |
| ΔPSQI | Adjusted | F = 1.143, *p* = 0.327 |
|  |  |  |

Unadjusted, the primary model including age, cumulative years of alcohol use, AUDIT total score, and number of prior inpatient detoxifications as covariates. Adjusted, the additionally adjusted model further including baseline variables that differed significantly among groups. Results were pooled across multiple imputation datasets using Rubin’s rules. AUDIT, Alcohol Use Disorders Identification Test; PHQ-9, Patient Health Questionnaire-9; GAD-7, Generalized Anxiety Disorder-7; PSQI, Pittsburgh Sleep Quality Index.

**Reference**

[1] Faul F, Erdfelder E, Buchner A, Lang AG. **Statistical power analyses using G*Power 3.1: Tests for correlation and regression analyses**. *Behavior Research Methods*. 2009;**41**:1149–1160.
